# Natural history of silent lacunar infarction: 10-year follow-up of a community-based prospective study of 0.5 million Chinese adults

**DOI:** 10.1016/j.lanwpc.2021.100309

**Published:** 2021-10-21

**Authors:** Zilong Hao, Yiping Chen, Neil Wright, Haiqiang Qin, Iain Turnbull, Yu Guo, Christiana Kartsonaki, Sam Sansome, PeiPei, Canqing Yu, Qijun Gu, Jianming Hu, Jun Lv, Liming Li, Ming Liu, Yongjun Wang, Robert Clarke, Zhengming Chen

**Affiliations:** 1 Center of Cerebrovascular Disease, Department of Neurology, West China Hospital, Sichuan University, Chengdu, China; 2 Medical Research Council Population Health Research Unit, (PHRU), Nuffield Department of Population Health, University of Oxford, Oxford, UK; 3 Clinical Trial Service Unit and Epidemiological Studies Unit (CTSU), Nuffield Department of Population Health, University of Oxford, Oxford, UK; 4 China National Clinical Research Centre for Neurological Disease; Beijing Institute for Brain Disorders; Beijing Key Laboratory of Translational Medicine for Cerebrovascular Disease; Department of Neurology, Beijing Tiantan Hospital, Capital Medical University, Beijing, China; 5 Chinese Academy of Medical Sciences, Dong Cheng District, Beijing, China; 6 Department of Epidemiology and Biostatistics, School of Public Health, Peking University Health Science Center, Beijing, China; 7 Tongxiang CDC, Tongxiang, Zhejiang, China; 8 Shimen Town Health Center, Tongxiang, Zhejiang, China

**Keywords:** Silent lacunar infarction, Cerebral small vessel disease, Stroke subtypes, Prognosis

## Abstract

**Background:**

Widespread use of brain imaging in China has resulted in an increased prevalence of silent lacunar infarct (LACI) in addition to symptomatic LACI, but their clinical relevance is not fully understood.

**Methods:**

We compared the 5-year risks of recurrent stroke and all-cause mortality for silent LACI vs symptomatic LACI in a prospective study of 489,597 Chinese adults with no history of stroke or ischemic heart disease at baseline. Data on recurrent stroke and all-cause mortality were obtained by linkage with local stroke and mortality registries and health insurance records for all hospital admissions.

**Findings:**

Among 12,150 cases with an adjudicated diagnosis of first-ever LACI, 4,134 (34%) had silent LACI and 8,016 (66%) had symptomatic LACI. All cases had brain imaging, but only 33% of silent LACI and 40% of symptomatic LACI cases had brain magnetic resonance imaging (MRI). The standardized event rates for silent LACI were 2-fold greater in urban than rural areas, but the ratios of silent LACI vs symptomatic LACI were similar in all areas. Cases with silent LACI vs symptomatic LACI had comparable 5-year risks of recurrent stroke (38% vs 43%) and all-cause mortality (11% vs 14%), respectively. For both silent and symptomatic LACI cases, most cases of recurrent stroke had non-LACI (70% vs 72%). While the relative risks of recurrent stroke did not differ by age, sex and area, the absolute risks of all-cause mortality varied by sex, age and area.

**Interpretation:**

The prognosis of cases with silent LACI was comparable with symptomatic LACI, and the results highlight the need for further randomized trials assessing the efficacy and safety of established treatments for ischemic stroke in cases with silent LACI.

**Funding:**

Wellcome Trust (212946/Z/18/Z, 202922/Z/16/Z, 104085/Z/14/Z, 088158/Z/09/Z) and National Key Research and Development Program of China (2016YFC0900500, 2016YFC0900501, 2016YFC0900504, 2016YFC1303904) and National Natural Science Foundation of China (91843302); UK Medical Research Council (MC_UU_00017/1,MC_UU_12026/2 MC_U137686851), Cancer Research UK (C16077/A29186; C500/A16896) and British Heart Foundation (CH/1996001/9454). ZH was supported West China Hospital, Sichuan University (ZYGD18009 and 2016YFC1300505) for a visiting scholarship to the University of Oxford, UK, during 2018-19.

**Research in Context**

***Evidence before this study:*** Silent lacunar infarcts (LACI) are small cerebral ischemic lesions (<15 mm diameter) detected on brain imaging in the territory of the deep perforating arteries and occurring in the absence of classical symptoms or signs of stroke. Little is known about the risks of recurrent stroke or all-cause mortality following silent LACI.

***Added value of this study:*** We compared the prognosis of silent LACI with symptomatic LACI, including 5-year risks of stroke recurrence and mortality, in a prospective study of 489 597 Chinese adults without stroke at baseline. Compared to participants with no confirmed stroke during follow-up, cases with silent LACI were older (60 vs 51 years) and had a higher prevalence of hypertension (19% vs 9%) and diabetes (10% vs 5%) at baseline. Cases with silent LACI vs symptomatic LACI had comparable 5-year risks of recurrent stroke (38% vs 43%) and all-cause mortality (11% vs 14%), respectively.

***Implications of all the available evidence:*** The long-term prognosis of cases with silent LACI was comparable with those with symptomatic LACI, prompting the need for further randomized trials assessing the efficacy and safety of established treatments for ischemic stroke in cases with silent LACI.

Alt-text: Unlabelled box

## 1 Introduction

Silent lacunar infarcts (LACI) are covert cerebral ischemic lesions that are detected on brain imaging examinations of individuals presenting with non-specific or atypical neurological symptoms without the classical features of an acute stroke [1]. LACI, or lacunes, are small brain infarcts (<15 mm in diameter), round or ovoid, that occur predominantly in the territory of the small deep perforating arteries of the subcortical regions of the brain and account about 20-35% of all ischemic strokes (IS) [1,2]. About 10% of LACI are found in the cortical regions of the brain. Most LACI result from small artery occlusion, but large artery atherosclerosis and cardiac embolism may also be involved [3], [4], [5]. Silent and symptomatic LACI are part of a spectrum of cerebral small vessel disease (CSVD), that includes white matter hyperintensities (WMH), perivascular spaces (PVS), cerebral microbleeds (CMB), and brain atrophy [6,7].

Magnetic resonance imaging (MRI) is more sensitive than computed tomography (CT) for detection of CSVD [7]. Patients with silent LACI may present with atypical neurological symptoms, including headache, atypical limb weakness or sensory loss or dizziness, but lack the typical features of stroke and the causal relevance of any presenting symptoms is uncertain. Previous studies of silent brain infarct (SBI) in developed countries have estimated prevalence rates of 10% to 28% [8,9]. Widespread use of brain imaging in China has resulted in increased detection of silent LACI in hospital settings [10,11], with estimated prevalence rates of 20%. While little is known about the prognosis of silent LACI vs symptomatic LACI in the same population, prior studies of SBI, have reported that about 10% of individuals developed a symptomatic stroke within 5 years, which was 2-fold greater than for individuals without SBI [2,3]. SBI has also been associated with increased risk of cognitive decline, psychiatric disorders and premature death [4], [5], [6], [7].

Previous studies have reported associations of hypertension, chronic kidney disease, metabolic syndrome and carotid artery disease with SBI [2,7,11], and identified elevated blood pressure as the chief modifiable cause of both silent and symptomatic CSCVD [2,7,11].The American Heart Association currently advocates primary stroke prevention strategies to reduce levels of vascular risk factors and maintain healthy lifestyles for the management and prevention of silent LACI [8]. Reliable evidence on the long-term prognosis of silent LACI cases could inform the design of clinical trials to evaluate the efficacy and safety of established treatments for ischaemic stroke in cases with silent LACI.

We compared the absolute risks, correlates and prognosis of incident cases of both silent LACI and symptomatic LACI in a 10-year follow-up of the China Kadoorie Biobank (CKB), a community-based prospective study of 0.5 million Chinese adults recruited in 2004-2008. The aims of this report were to: (i) compare the distribution and determinants of silent vs symptomatic LACI; (ii) assess the 5-year risks of recurrent stroke and all-cause mortality in silent vs symptomatic LACI; and (iii) examine the pathological types of recurrent stroke in silent vs symptomatic LACI.

## 2 Methods

### 2.1 Study population

Details of the study design and participants in the CKB study have been previously described (9,10). Briefly, CKB is an ongoing community-based prospective study, involving a total of 512,726 participants, aged 30-79 years, who were recruited from 10 geographically diverse areas (5 urban and 5 rural) in China between 2004 and 2008. Data were collected at baseline by trained health workers using interviewer-administered laptop-based questionnaires. Moreover, a range of physical measurements were also recorded, and a blood sample was collected for long-term storage. Local, national and international ethical approvals were obtained and all participants provided written informed consent.

### 2.2 Data collection

The questionnaire data collected included questions about demographic and socioeconomic factors (e.g. date of birth, sex, marital status, education, household income), lifestyle factors (e.g. smoking, alcohol drinking, diet, and physical activity), and personal and family medical history. Blood pressure was measured twice (or three times if the difference between the first two measurements was >10 mm Hg for SBP) using a UA-779 digital sphygmomanometer (A&D Instruments; Abingdon, UK) after participants had been seated in the resting position for at least 5 minutes, with the mean of the last two measurements used for analyses.

### 2.3 Follow up and case ascertainment

Data on all hospital admissions or deaths were obtained by linkage via unique national identification numbers to cause-specific mortality and morbidity registers and to the national health insurance database. In CKB study, follow-up for almost 97% of study participants has been obtained by linkage to the national health insurance claims database and mortality registers, and follow-up for participants who were uninsured was obtained by visits to households and active screening of disease and death registries to identify hospitalizations for stroke, other vascular diseases and deaths.

The medical records of any hospitalised cases of first incident stroke were retrieved periodically, and the hospital discharge diagnoses were adjudicated using bespoke web-based software by Chinese neurologists blinded to other information recorded at enrolment in CKB [11]. Causes of death, obtained from official death certificates, were supplemented by available medical records. In the small proportion of deaths (<5%) occurring without recent medical attention in hospital, standard procedures were used to determine probable causes of death based on symptoms and signs provided by informants (usually family members). By 1 January 2018, a total of 49,459 (9.6%) participants had died and 5,302 (1%) were lost to follow-up.

Stroke was defined as an acute-onset focal neurological deficit of presumed vascular origin lasting more than 24 hours. The present analyses were restricted to individuals with available data on brain imaging results. Cases of IS (ICD-10 codes I63) were classified into LACI and non-LACI stroke types using clinical and brain imaging findings. LACI was defined as a stroke with brain imaging evidence of single or multiple lesions that each were <15 mm in diameter on CT or MRI. Where multiple lesions were found, cases were classified as non-LACI if a single lesion was >15 mm. If both LACI and non-LACI co-existed, cases were classified as non-LACI (**Supplementary Figure 1**). In addition, cases were classified as non-LACI if typical focal neurological deficits were found or an IS type was unspecified. Hence, any IS cases where LACI were excluded, was defined as non-LACI. Silent LACI was defined using the following criteria: (i) abnormal signal intensity in a vascular distribution of <15 mm in diameter on CT or MRI; and (ii) no focal neurological signs or symptoms corresponding to cerebral ischemic lesions including lacunar syndromes.

Ischemic heart disease (IHD) is a frequent co-morbidity with stroke. In the CKB study, all prior IHD cases were self-reported doctor-diagnosed cases. We cannot exclude the possibility that some IHD cases might also have had a stroke which would affect the prognosis of silent vs symptomatic LACI. Hence, we also excluded participants with prior IHD in the present analyses. Finally, among 21,410 adjudicated cases of first-ever IS with no prior history of stroke or transient ischemic attack (TIA) or IHD at baseline and complete data on brain imaging, the present report compared the outcomes of 8016 symptomatic LACI cases with 4134 silent LACI cases (**Supplementary Figure 1**). The main outcome measures included recurrent stroke or all-cause mortality following their initial diagnosis with first-ever silent LACI or symptomatic LACI.

### 2.4 Statistical analysis

Selected characteristics at baseline were presented for participants who had silent LACI, or symptomatic LACI. Data are presented as means and standard deviations (SD) for continuous variables and proportions for categorical variables after adjusting for age at baseline, sex and study area. Age-standardized event rates of silent LACI and the cumulative risks of recurrent stroke and all-cause mortality were calculated, and compared with those for individuals diagnosed with symptomatic LACI. Cumulative mortality rates following a first incident LACI were calculated as one minus Kaplan–Meier survival probability, with censoring at death or date of last follow-up. The cumulative event rates for recurrent stroke and other CVD outcomes were calculated by the cumulative incidence function, treating death from any cause as a competing risk. The cumulative incidence of competing risks were estimated using the cmprsk package in R and the corresponding 95% confidence intervals were based on the log-minus-log transformation [12,13]. The event rates were estimated separately by stroke type, and by sex, area (urban or rural), and age at first stroke (>65 years or 65+ years). In analyses of recurrent strokes classified by type, different stroke types were treated as competing risks so that the analysis includes only the first recurrent stroke for each participant. Unless otherwise specified, recurrent cases of stroke, major vascular events or deaths were restricted to those occurring after 28 days following a first stroke event. Cox proportional hazards models were used to estimate hazard ratios (HRs) and 95% confidence intervals (CIs) for the associations of silent LACI and symptomatic LACI with usual levels of systolic blood pressure (SBP). Models were stratified by 5-year age-at-risk groups, sex, region, and adjusted for education (4 groups), and alcohol consumption (3 groups). The HRs were corrected for regression dilution bias using the McMahon-Peto method. The HRs and 95% CIs for groups of SBP were estimated using group-specific variances, such that the HR in each group, including the reference group, were associated with a group-specific 95% CI. All analyses were performed using R version 3.6.2.

### 2.5 Role of the Funding Source

The funding organizations had no role in the design, conduct, analysis, interpretation, or approval for the submission of this report.

## 3 Results

Among the 12,150 cases with a clinically-adjudicated diagnosis of LACI, 4,134 (34%) had silent LACI and 8,016 (66%) had symptomatic LACI. Cases with silent LACI had comparable baseline characteristics as those with symptomatic LACI, but were more likely to be female (61% vs 54%), and less likely to have diabetes (10% vs 13%) or hypertension (19% vs 22%) **(**Table 1**)**. Compared to participants without stroke during follow-up, cases with silent LACI were older (60 vs 51 years) and had a higher prevalence of hypertension (19% vs 9%) and diabetes (10% vs 5%) at baseline (Table 1).

**Table 1:** Baseline characteristics of participants by subtypes of first adjudicated ischemic stroke

	Silent LACI (n = 4,134)	Symptomatic LACI (n = 8,016)	No confirmed stroke (n = 464,114)
Age at baseline, years, %			
30 - 39	2.6	2.2	16.5
40 - 49	16.8	16.7	31.6
50 - 59	34.1	33.7	30.6
60 - 69	32.2	32.4	15.9
70 - 79	14.2	15.0	5.3
Mean (SD)	60.2 (9.5)	59.6 (9.5)	51.2 (10.4)
Age at first confirmed stroke, years	67.2 (9.3)	66.5 (9.5)	
Female, %	61.2	53.8	59.4
Rural, %	39.6	40.0	57.3
Married, %	85.5	86.0	91.1
Household income <10,000 CNY/y, %	30.6	29.0	28.2
No formal school, %	21.1	19.2	18.5
Prior disease, %			
Hypertension	19.2	21.8	9.2
Diabetes	9.9	13.1	5.0
Ever regular smoker, %*			
Men	68.4	73.8	74.5
Women	5.7	5.8	3.0
Ever regular drinker, %⁎⁎			
Men	40.9	44.1	41.8
Women	3.3	2.6	2.9
Physical activity, MET-h/day	20.0 (11.5)	20.5 (12.0)	21.6 (13.9)
SBP, mmHg	137.6 (23.0)	140.8 (24.2)	130.1 (20.6)
DBP, mmHg	81.3 (11.8)	83.0 (12.3)	77.4 (11.0)
BMI, kg/m²	24.2 (3.4)	24.4 (3.5)	23.6 (3.3)
Waist:Hip ratio	0.89 (0.07)	0.90 (0.07)	0.88 (0.07)
Imaging type, %			
CT only	67.5	59.6	
MRI	32.5	40.4	

Values are mean (SD) unless otherwise stated. Means and percentages are directly standardised to age, sex and study area structure of the CKB study population after excluding 23,129 participants with prior CHD or stroke/TIA.

MET-h/day = Metabolic equivalents of task hours per day; SBP = Systolic blood pressure; DBP = Diastolic blood pressure; BMI = Body mass index

⁎ Current or ex-regular smoker

⁎⁎ Ex-regular, reduced intake or weekly alcohol drinking

The age-standardized event rates of silent LACI were higher in urban than in rural areas (104 vs 54 per 100,000 person-years), as were the rates of symptomatic LACI (**Supplementary Figure 2**). Across the 10 study areas, the absolute rates of silent LACI varied over 10 to 20-fold from 15 (Zhejiang, a rural area in south East of China) to 288 per 100,000 person-years (Harbin, an urban area in North East of China). Similar regional variations were observed for the standardized event rates for symptomatic LACI (**Supplementary Figure 2**). Despite substantial variations in the absolute event rates, the ratios of silent versus symptomatic LACI were comparable in all 10 study areas (**Supplementary Figure 2**). Details of the presenting symptoms of silent LACI cases recorded in all 4134 silent LACI cases are provided in **Supplementary Table 1** and these included dizziness or headache (80.3%), bilateral limb weakness or four-limb weakness or numbness in 9.2%, and coma, drowsiness or clumsiness in 2.3%.

**Figure 1:**
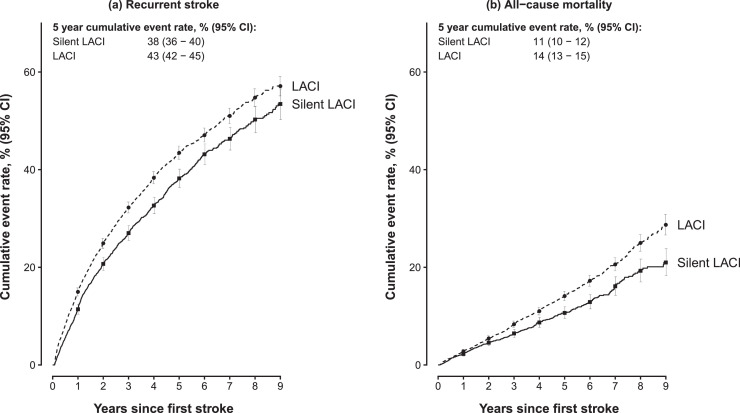
Cumulative event rate of recurrent stroke and all−cause mortality from 28 days after onset of first ischemic stroke event Lines indicate the cumulative incidence function, starting at 28 days after the onset of first stroke, separately for silent LACI and symptomatic LACI by years of follow up. Deaths from any cause were treated as competing risks. Participants experiencing an event or death within the first 28 days from first stroke were excluded

The case fatality rates at 28 days for silent LACI were 0.5%, which were similar to those with symptomatic LACI (0.8%; **Supplementary Table 2**). In both cases, the case fatality rates did not differ materially by gender or area, but increased progressively with age (**Supplementary Table 2**). Among survivors of silent LACI at 28 days, the cumulative all-cause mortality rates were 2%, 11% and 21% at 1, 5, 9 years, which were slightly lower than that observed (3%, 14% and 29%, respectively) among those with symptomatic LACI (Table 2 and Figure 1). As shown in **Supplementary Figure 3**, the all-cause mortality rates of silent LACI at 5 years were 2-fold greater in men vs women (14% vs 8%), and in rural vs urban residents (15% vs 8%) and were 3-fold greater in individuals over 65 years vs under 65 years (16% vs 5%). In contrast, the cumulative all-cause mortality rates at 1, 5 and 9 years in individuals without any stroke were 0%, 3% and 6%, respectively, which were less than one-third of those observed among cases with silent LACI (**Supplementary Table 3**).

**Table 2:** Cumulative event rate of recurrent stroke and all-cause mortality from 28 days after first ischemic stroke subtype

		Years since first IS									
LACI		28 days	1	2	3	4	5	6	7	8	9
Silent	Recurrent stroke										
	No. events	0	451	786	963	1,089	1,183	1,241	1,268	1,290	1,300
	No. free of any events and deaths	3,957	3,446	2,417	1,712	1,218	816	508	322	172	91
	Cumulative event rate, % (95% CI)	0	11 (10-12)	21 (19-22)	27 (26-29)	33 (31-34)	38 (36-40)	43 (41-45)	46 (44-49)	50 (48-53)	53 (50-57)
	All-cause mortality										
	No. events	0	93	178	231	280	313	338	363	380	385
	No. free of any events and deaths	4,115	4,017	3,052	2,337	1,774	1,243	824	546	313	167
	Cumulative event rate, % (95% CI)	0	2 (2-3)	5 (4-5)	6 (6-7)	9 (8-10)	11 (10-12)	13 (11-14)	16 (14-18)	19 (17-22)	21 (18-24)
Symptomatic	Recurrent stroke										
	No. events	0	1,119	1,806	2,224	2,504	2,687	2,780	2,851	2,897	2,916
	No. free of any events and deaths	7,475	6,230	4,511	3,309	2,305	1,539	956	596	345	185
	Cumulative event rate, % (95% CI)	0	15 (14-16)	25 (24-26)	32 (31-33)	38 (37-40)	43 (42-45)	47 (46-48)	51 (49-53)	55 (53-57)	57 (55-59)
	All-cause mortality										
	No. events	0	222	414	586	718	834	917	979	1,035	1,064
	No. free of any events and deaths	7,945	7,708	6,225	5,010	3,789	2,706	1,807	1,214	761	420
	Cumulative event rate, % (95% CI)	0	3 (2-3)	5 (5-6)	8 (8-9)	11 (10-12)	14 (13-15)	17 (16-18)	21 (19-22)	25 (23-27)	29 (27-31)

Among survivors (who were alive and had no recurrent stroke) at 28 days after diagnosis of first silent LACI, the cumulative recurrence rates were 11%, 38% and 53% at 1, 5, and 9 years, respectively, which were slightly lower than 15%, 43% and 57% among those with symptomatic LACI (Table 2 and Figure 1**)**. The stroke recurrence rates were similar between silent and symptomatic LACI cases. The all-cause mortality rates for both LACI types were higher in men than women, among urban than rural residents, and in those aged over 65 than under 65 years at first incident stroke (**Supplementary Figure 3**). In contrast, the cumulative event rates of first stroke in individuals without stroke at baseline were 2% and 4% at 5 and 9 years (and the death rates were 3% and 6%, respectively).

**Figure 2:**
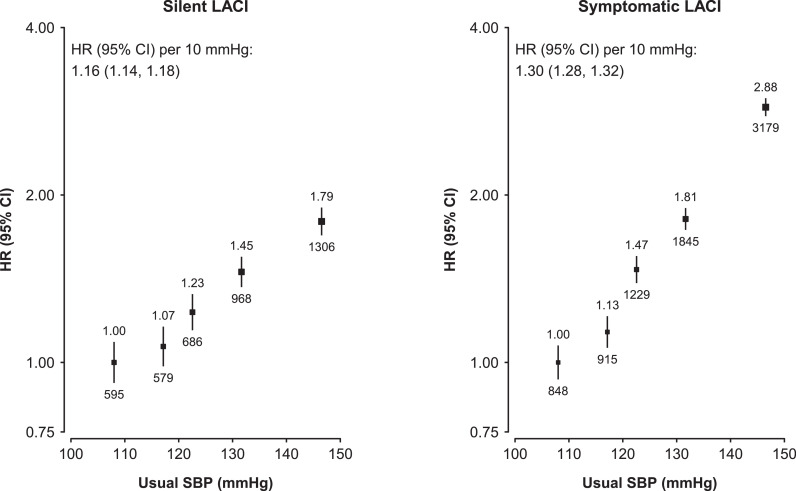
Associations of usual SBP with risk of silent and symptomatic LACI Cox proportional hazards models were used to estimate the HR (95% CI) of silent LACI and symptomatic LACI per 10 mmHg higher usual levels of systolic blood pressure (SBP). The models were adjusted for age at baseline, sex, and area. For each category, the area of each square is inversely proportional to the variance of the category-specific log risk, which also determines the 95% CI. The HRs are shown above each square and the number of events are shown under each square.

Following a first silent LACI, 70% of all the recurrent strokes at 5 years after initial diagnosis had non-LACI, only 24% also had LACI, 5% had ICH and 2% had unspecified stroke **(**Table 3**)**. The patterns were similar for symptomatic LACI with corresponding rates of 72%, 19%, 5% and 3%, respectively **(**Table 3**)**. Both silent and symptomatic LACI showed strong positive and log-linear associations with higher levels of systolic blood pressure (SBP), with adjusted HR per 10 mmHg higher usual SBP of 1.16 (95% CI 1.14-1.18) and 1.30 (1.28-1.32), respectively **(**Figure 2**).**

**Table 3:** Cumulative event rates of further stroke by types from 28 days after first diagnosis of LACI

	Years since first diagnosis										
LACI	Type of recurrent stroke	1	2	3	4	5	6	7	8	9	
Silent	LACI	No. events	90	177	218	254	278	293	299	305	308
		Cumulative event rate, % (95% CI)	2 (2-3)	5 (4-5)	6 (5-7)	8 (7-9)	9 (8-10)	10 (9-12)	11 (10-13)	12 (11-14)	13 (11-15)
		Proportion of recurrent strokes, %	20	23	23	24	24	24	24	24	24
	Non-LACI	No. events	334	563	689	767	832	871	891	903	910
		Cumulative event rate, % (95% CI)	8 (8-9)	15 (14-16)	19 (18-21)	23 (21-24)	27 (25-28)	30 (28-32)	32 (30-34)	35 (32-37)	37 (34-40)
		Proportion of recurrent strokes, %	74	72	71	70	70	70	70	69	69
	ICH	No. events	17	31	40	47	52	54	55	57	57
		Cumulative event rate, % (95% CI)	0 (0-1)	1 (1-1)	1 (1-2)	1 (1-2)	2 (1-2)	2 (1-2)	2 (1-3)	2 (2-3)	2 (2-3)
		Proportion of recurrent strokes, %	4	4	4	4	5	4	4	5	4
	Unspecified	No. events	10	15	16	21	21	23	23	25	25
		Cumulative event rate, % (95% CI)	0 (0-0)	0 (0-1)	0 (0-1)	1 (0-1)	1 (0-1)	1 (1-1)	1 (1-1)	1 (1-2)	1 (1-2)
		Proportion of recurrent strokes, %	2	2	2	2	2	2	2	2	2
	All stroke	No. events	451	786	963	1,089	1,183	1,241	1,268	1,290	1,300
		Cumulative event rate, % (95% CI)	11 (10-12)	21 (19-22)	27 (26-29)	33 (31-34)	38 (36-40)	43 (41-45)	46 (44-49)	50 (48-53)	53 (50-57)
Symptomatic	LACI	No. events	179	320	410	468	508	530	552	557	561
		Cumulative event rate, % (95% CI)	2 (2-3)	4 (4-5)	6 (5-7)	7 (7-8)	8 (8-9)	9 (8-10)	10 (10-11)	11 (10-12)	11 (10-13)
		Proportion of recurrent strokes, %	16	18	19	19	19	20	21	20	20
	Non-LACI	No. events	849	1,341	1,633	1,829	1,950	2,016	2,059	2,094	2,104
		Cumulative event rate, % (95% CI)	11 (11-12)	18 (18-19)	24 (23-25)	28 (27-29)	31 (30-33)	34 (33-35)	36 (35-38)	39 (37-41)	40 (39-42)
		Proportion of recurrent strokes, %	76	74	73	73	72	72	71	71	71
	ICH	No. events	52	85	113	129	141	144	148	153	157
		Cumulative event rate, % (95% CI)	1 (1-1)	1 (1-1)	2 (1-2)	2 (2-2)	2 (2-3)	2 (2-3)	3 (2-3)	3 (3-4)	4 (3-4)
		Proportion of recurrent strokes, %	5	5	5	5	5	5	5	6	6
	Unspecified	No. events	39	60	68	78	88	90	92	93	94
		Cumulative event rate, % (95% CI)	1 (0-1)	1 (1-1)	1 (1-1)	1 (1-1)	1 (1-2)	2 (1-2)	2 (1-2)	2 (1-2)	2 (1-2)
		Proportion of recurrent strokes, %	3	3	3	3	3	3	3	3	3
	All stroke	No. events	1,119	1,806	2,224	2,504	2,687	2,780	2,851	2,897	2,916
		Cumulative event rate, % (95% CI)	15 (14-16)	25 (24-26)	32 (31-33)	38 (37-40)	43 (42-45)	47 (46-48)	51 (49-53)	55 (53-57)	57 (55-59)

## 4 Discussion

This large community-based prospective cohort study confirmed previously reported high incidence rates of total stroke in Chinese adults. A high proportion of reported IS cases were independently adjudicated by local neurologists as silent or covert LACI. Although the absolute rates of silent LACI varied by 10-fold between study areas (consistent with rates for other IS types), the ratios of silent LACI vs symptomatic LACI were similar in each area. Among silent LACI cases, about 4 in 10 had a recurrent stroke and about 1 in 10 died by 5 years consistent with event rates for symptomatic LACI. For individuals with silent LACI who experienced a recurrent stroke type, two-thirds had non-LACI (usually with poor prognosis) and only a quarter had LACI, consistent with those for symptomatic LACI. While the risks of recurrent stroke at 5 years did not differ by age, sex and area, the all-cause mortality rates were higher in men, in rural areas and in those aged over 65 years.

The absence of consensus on diagnostic criteria or type of imaging used for diagnosis for silent LACI and SBI has complicated comparisons between studies conducted in different populations. The MRI methodology (including magnetic field strength, slice thickness, and gap between slices) has varied between studies [14], and hence, the absolute rates of silent SBI have varied by over 10-fold (from 5% to 62%). In the present study, about one-fifth of IS cases had silent LACI. Although there were over 10-fold differences in the age-standardized admission rates of silent LACI between the 10 study areas, the ratios of silent LACI vs symptomatic LACI were similar in each area, suggesting that regional discrepancies reflected differences in disease rates rather than differences in diagnostic practice.

Previous studies conducted in mainly Western populations of silent LACI or SBI cases, have been constrained by small sample size and included comparisons with healthy individuals rather than symptomatic LACI cases. In a meta-analysis of >14,000 participants from 13 cohort studies with a mean follow-up duration of 2-14.5 years, individuals with SBI detected by MRI had 2-fold higher risks of subsequent stroke compared with those without SBI [3], but no comparisons were provided for cases with symptomatic IS. In the Cardiovascular Heart Study of 923 participants with an MRI diagnosis of SBI, 7.3% had symptomatic strokes during 4-year follow-up, with a 50% higher risk than those without SBI [15]. In the Rotterdam Scan Study, in which 217 cases of SBI were detected using MRI among 1,007 participants, 11.7% had subsequent stroke at 4-years, rising to 23.7% at 10-years [16,17].

The present study is the first large study to compare the prognosis of silent LACI and symptomatic LACI cases in the same study population, and demonstrated that the long-term event rates of subsequent stroke and death were similar for both silent and symptomatic LACI. Moreover, the stroke recurrence rates were higher than those reported in previous studies of SBI in Western populations. The 5-year risks of all-cause mortality for silent LACI in the present study were comparable with those in cases with a hospital diagnosis of TIA or minor stroke (10.6% risk of all-cause mortality) in Western countries [18]. The reasons for the poor long-term prognosis among silent LACI cases in China are unclear, but may reflect differences in study design and disease diagnosis, or lower compliance (or intensity of use) of effective treatments after diagnosis. Previous reports had suggested that up to 50% of stroke cases in China discontinued medication for secondary prevention of stroke by 3 months after hospital discharge and that hypertension was poorly managed following stroke [19,20].

Few previous studies have examined the long-term prognosis of silent LACI cases, or assessed stroke recurrence by pathological type and IS type in silent and symptomatic LACI cases. In the US Cardiovascular Heart Study, half of all recurrent strokes that occurred among individuals with SBI had cardio-embolic strokes, followed by lacunar and atherosclerotic strokes [15]. However, the number of recurrent stroke cases were too small (only 47 cases) to provide reliable evidence on outcomes. With complete follow-up for 10 years after enrolment, the present study had a greater number of recurrent stroke cases than most previous studies and demonstrated that most recurrent stroke cases following both silent and symptomatic LACI were non-LACI. There is evidence that ipsilateral large-artery atherosclerosis and cardio-embolism are independent predictors of recurrent stroke in cases of TIA or minor stroke [18]. Hence, more intensive treatment of the underlying causes of silent LACI is required, including screening for atrial fibrillation or large-artery atherosclerosis. Such strategies may be particularly relevant in individuals at high-risk of recurrent stroke (e.g., men, rural residents and individuals aged over 65 years). Moreover, the strong log-linear positive associations of higher levels of SBP with higher risks of both silent and symptomatic LACI highlight the potential importance of strategies to lower blood pressure for prevention of both silent LACI and symptomatic LACI [2,11]. The comparable results for associations of both silent and symptomatic LACI with elevated levels of SBP reflects shared risk factors, in addition to comparable outcomes. Consequently, it is likely that treatment approaches for silent LACI will be similar to those for symptomatic LACI. However, further randomized evidence is needed to assess the efficacy and safety of established treatments for IS (including antiplatelet agents and statins) in cases with silent LACI.

The chief strengths of the present study include a prospective design and large number of adjudicated cases with silent LACI. Moreover, cases with silent LACI were detected in a clinical setting rather than by imaging detection of all study participants in a research setting (using brain imaging to detect abnormalities in all study participants). Hence, the present study was able to compare the prognosis of silent LACI vs symptomatic LACI. However, the present study also had several limitations. Firstly, direct visualization of brain imaging for IS cases was not possible and features of ischemic lesions like size (<15 mm) and location were only available from radiological reports. Consequently, we were unable to distinguish lacunar lesions from other types of small vessel disease. Since the diagnosis of symptomatic LACI was prioritized in individuals with both symptomatic and silent LACI cases, it was not possible to compare the prognosis of symptomatic LACI with and without a coexisting diagnoses of silent LACI. Secondly, only 38% of total silent and symptomatic LACI cases were assessed by MRI, which is more sensitive than CT. Consequently, the reported incident events rates for first LACI and silent LACI are likely to have underestimated the true incidence rates in this study population. Nevertheless, analyses of the cumulative event rates for stroke and all-cause mortality after stratification by type of brain imaging used (MRI or CT only) demonstrated no material differences in survival rates by those detected by CT only vs MRI **(Supplementary Figure 5).** Thirdly, the findings cannot be generalizable to the Chinese population, as the CKB study was designed to investigate the causes of diseases in diverse regions of China rather than being representative of the Chinese population. Participants were not screened systematically for silent LACI using MRI and, hence, the standardized event rates for silent LACI should be interpreted with caution when making national and international comparisons. The standardized event rates of silent LACI and LACI were higher in urban than in rural areas, which may reflect the greater availability of MRI brain imaging and better access to hospitals in urban areas, but the ratios of silent LACI vs symptomatic LACI were similar in each area (and in rural and urban areas), which suggests that any area differences reflect differences in disease rates rather than differences in diagnostic practice. However, detailed assessment of the causes of regional differences in silent vs symptomatic LACI incidence rates requires further study. Fourthly, outcome ascertainment was dependent on linkage to coded hospital admissions and death certification data and recurrent stroke events were not adjudicated. Stroke recurrence could not be distinguished from stroke progression or from stroke as a co-morbidity of another primary diagnosis. In addition, it is possible that patients with silent LACI may have represented more frequently with atypical symptoms requiring definitive diagnosis while those with symptomatic LACI whose symptoms were minor may have been missed by follow-up. Hence, the possibility of overestimation or underestimation of stroke recurrence after a first-ever stroke cannot be entirely excluded. Finally, the results cannot be generalized to the overall Chinese population for primary prevention in the absence of agreed diagnostic criteria for silent or covert LACI using CT, which is far more widely used than MRI for brain imaging in Chinese hospitals. Hence, the results of this study are more likely to be generalizable for future observational studies for screening or clinical trials assessing the efficacy and safety of treatments for silent LACI cases. Moreover, there is no agreement on the relevance, if any, of the associated non-specific neurological symptoms in cases with silent LACI. Future studies should compare the distribution, risk factors and prognosis of silent LACI cases with other stroke cases of undetermined etiology (i.e., cryptogenic etiology, consensus or non-consensus TIA) [21].

Overall, the present study demonstrated that the absolute rates of silent LACI in Chinese adults were high and that their long-term prognosis was poor, and consistent with the findings for symptomatic LACI. Moreover, about two-thirds of recurrent strokes among silent LACI cases were non-LACI, suggesting a shared etiology. Further studies are required to assess the efficacy and safety of treatments for silent LACI and to evaluate screening approaches for silent LACI in high-risk populations once effective treatments have been approved.

## 5 Contributions

ZH wrote the first draft of the report and conducted the initial statistical analyses. NW conducted the additional statistical analyses and YC, RC and ZC supervised the analyses and revised the report. YC, HQ designed bespoke web-based systems for clinical adjudication and IT and ZH conducted central review of all silent LACI cases in Oxford. YC, ZC, RC and LL had full access to the data. All authors were involved in study design, conduct, long-term follow-up, analysis of data, and provided critical comments on the final version of the report. RC and YC had final responsibility for the decision to submit the report for publication.

## Declaration of Competing Interest

We declare that we have no conflicts of interest or financial disclosures.

## Appendix Supplementary materials

Image, application 1
