# Supplementary material for: Natural history of silent lacunar infarction: 10-year follow-up of a community-based prospective study of 0.5 million Chinese adults
Source: Lancet Reg Health West Pac. 2021 Oct 21;17:100309. doi: 10.1016/j.lanwpc.2021.100309 (PMC8551852; doi:10.1016/j.lanwpc.2021.100309)

**Supplementary material for “Natural history of silent lacunar infarction: 10-year follow-up of a community-based prospective study of 0.5 million Chinese adults”**

**Table of Contents**

[Supplementary Table 1: Presenting symptoms of 4134 silent LACI cases classified by duration of symptoms 2](#_Toc70585708)

[Supplementary Table 2: Case fatality rates within 28 days after first diagnosis of LACI 3](#_Toc70585709)

[Supplementary Table 3: Cumulative event rate of first stroke and all-cause mortality from baseline 5](#_Toc70585710)

[Supplementary Figure 1 Flow sheet of study participants 6](#_Toc70585711)

[Supplementary Figure 2: Age−standardised event rate of silent LACI and LACI across 10 regions 7](#_Toc70585712)

[Supplementary Figure 3: Cumulative all−cause mortality rate from 28 days after first ischemic stroke of different subtypes by (a) sex, (b) area and (c) age 8](#_Toc70585713)

[Supplementary Figure 4: Cumulative event rate of recurrent stroke from 28 days after first ischemic stroke of different subtypes by (a) sex, (b) area and (c) age 9](#_Toc70585714)

Supplementary Figure 5: Cumulative event rate of (a) recurrent stroke and (b) all−cause mortality from 28 days after onset of first ischemic stroke event by imaging type ……………………….....10

# Supplementary Table 1: Presenting symptoms of 4134 silent LACI cases classified by duration of symptoms

| **Presenting symptoms** | **Duration (%) <24 hours** | | **Duration (%)**  **>24 hours<28 days** | **Duration (%)**  **>28 days** | **Total (%)** |
| --- | --- | --- | --- | --- | --- |
| Dizziness/headache | 788 (19.1) | | 2217 (53.6) | 316 (7.6) | 3321 (80.3) |
| Unilateral weakness or numbness | 6 (0.1) | | 25 (0.6) | 6 (0.1) | 37 (0.9) |
| Bilateral (or four limb) weakness or numbness | 79 (1.9) | | 254 (6.1) | 47 (1.1) | 380 (9.2) |
| Coma/unconscious/drowsy/clumsy | 47 (1.1) | | 40 (1.0) | 8 (0.2) | 95 (2.3) |
| Unsteady walking | 17 (0.4) | | 57 (1.4) | 11 (0.3) | 85 (2.1) |
| Language difficulty | 17 (0.4) | | 28 (0.7) | 2 (0.0) | 47 (1.1) |
| Memory loss | 2 (0.0) | | 26 (0.6) | 15 (0.4) | 43 (1.0) |
| *Other symptoms (non-central nervous system) | 33 (0.8) | | 75 (1.8) | 18 (0.4) | 126 (3.0) |
| **Total** | **989** | | **2722** | **423** | **4134** |
| * Vomiting after eating, Nausea, vomiting, Palpitations, etc | | |  |  |  |

# Supplementary Table 2: Case fatality rates within 28 days after first diagnosis of LACI

|  | **Silent LACI** | | **Sympomatic LACI** | |
| --- | --- | --- | --- | --- |
|  | **Deaths / Cases** | **% (95% CI)** | **Deaths / Cases** | **% (95% CI)** |
| All | 19 / 4,134 | 0.5 (0.3-0.7) | 68 / 8,016 | 0.8 (0.7-1.1) |
| Age at stroke, years |  |  |  |  |
| 30-39 | 0 / 6 | 0.0 (0.0-45.9) | 0 / 15 | 0.0 (0.0-21.8) |
| 40-49 | 1 / 203 | 0.5 (0.0-2.7) | 1 / 417 | 0.2 (0.0-1.3) |
| 50-59 | 3 / 957 | 0.3 (0.1-0.9) | 3 / 1,888 | 0.2 (0.0-0.5) |
| 60-69 | 2 / 1,486 | 0.1 (0.0-0.5) | 14 / 2,752 | 0.5 (0.3-0.9) |
| 70-79 | 10 / 1,271 | 0.8 (0.4-1.4) | 42 / 2,554 | 1.6 (1.2-2.2) |
| 80-89 | 3 / 211 | 1.4 (0.3-4.1) | 8 / 390 | 2.1 (0.9-4.0) |
| *Trend:* |  | *p = 0.03* |  | *p < 0.01* |
| Men | 9 / 1,602 | 0.6 (0.3-1.1) | 38 / 3,705 | 1.0 (0.7-1.4) |
| Women | 10 / 2,532 | 0.4 (0.2-0.7) | 30 / 4,311 | 0.7 (0.5-1.0) |
| *Heterogeneity:* |  | *p = 0.59* |  | *p = 0.14* |
| Rural | 11 / 1,636 | 0.7 (0.3-1.2) | 34 / 3,210 | 1.1 (0.7-1.5) |
| Urban | 8 / 2,498 | 0.3 (0.1-0.6) | 34 / 4,806 | 0.7 (0.5-1.0) |
| *Heterogeneity:* |  | *p = 0.16* |  | *p = 0.12* |

# Supplementary Table 3: Cumulative event rate of first stroke and all-cause mortality from baseline

|  | **Years from baseline** | | | | | | | | | |
| --- | --- | --- | --- | --- | --- | --- | --- | --- | --- | --- |
|  | **0** | **1** | **2** | **3** | **4** | **5** | **6** | **7** | **8** | **9** |
| No. strokes | 0 | 725 | 1,816 | 3,258 | 5,059 | 7,361 | 10,136 | 13,414 | 17,024 | 20,430 |
| Cumulative event rate for all stroke, % (95% CI) | 0 | 0 | 0 | 1 (1-1) | 1 (1-1) | 2 (1-2) | 2 (2-2) | 3 (3-3) | 3 (3-4) | 4 (4-4) |
| No. deaths^*^ | 0 | 1,877 | 4,407 | 7,242 | 10,275 | 13,570 | 16,989 | 20,459 | 24,143 | 28,143 |
| Cumulative all-cause mortality rate, % (95% CI)^*^ | 0 | 0 | 1 (1-1) | 1 (1-2) | 2 (2-2) | 3 (3-3) | 4 (3-4) | 4 (4-4) | 5 (5-5) | 6 (6-6) |
| No. free of any stroke and deaths | 489,597 | 486,944 | 483,272 | 478,901 | 474,030 | 468,428 | 461,956 | 454,501 | 446,440 | 437,948 |
| ^*^ Censored at first stroke, so that this represents the cumulative event rate in the stroke-free population. | | | | | | | | | | |

# Supplementary Figure 1: Flow sheet of study participants


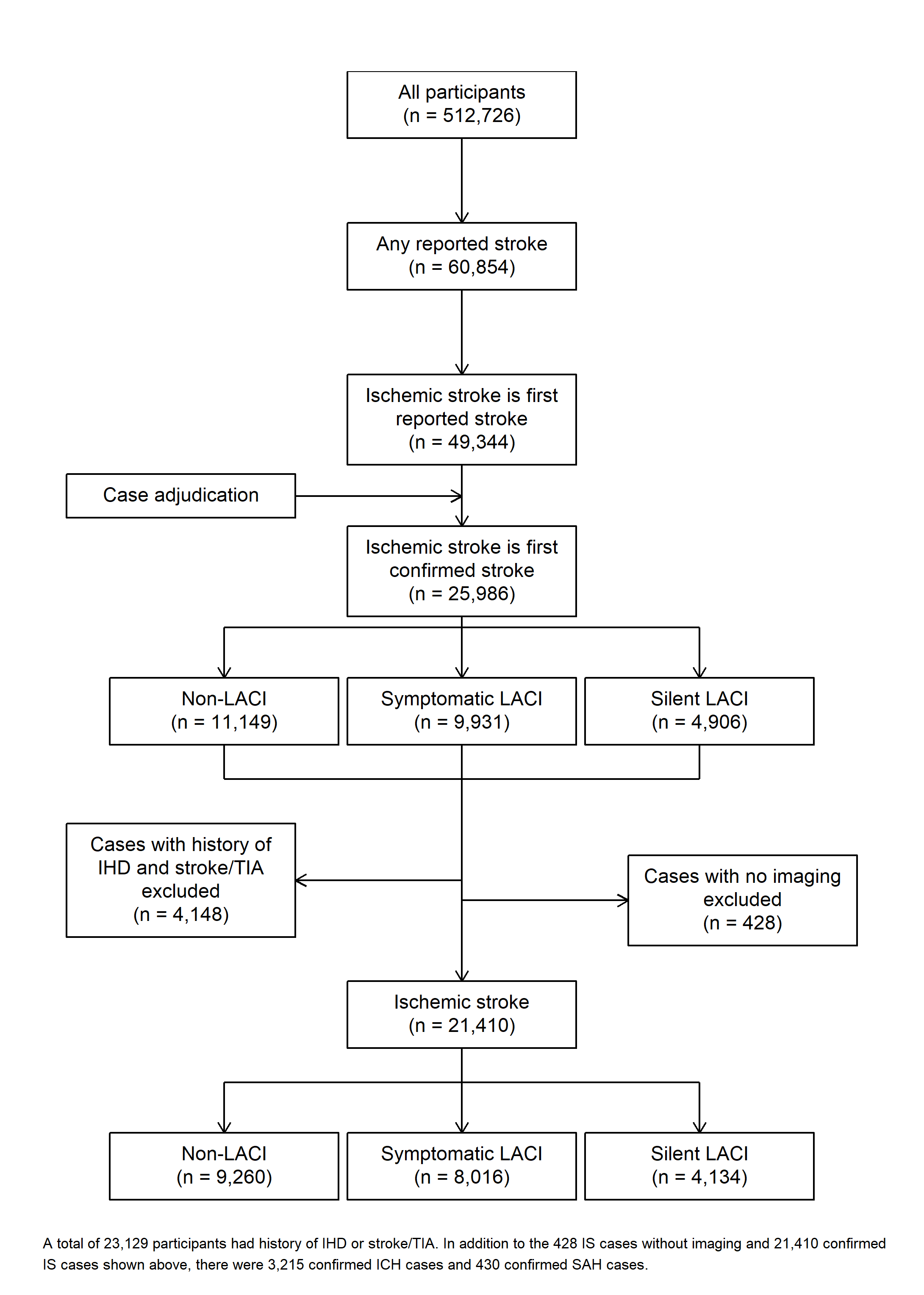


#
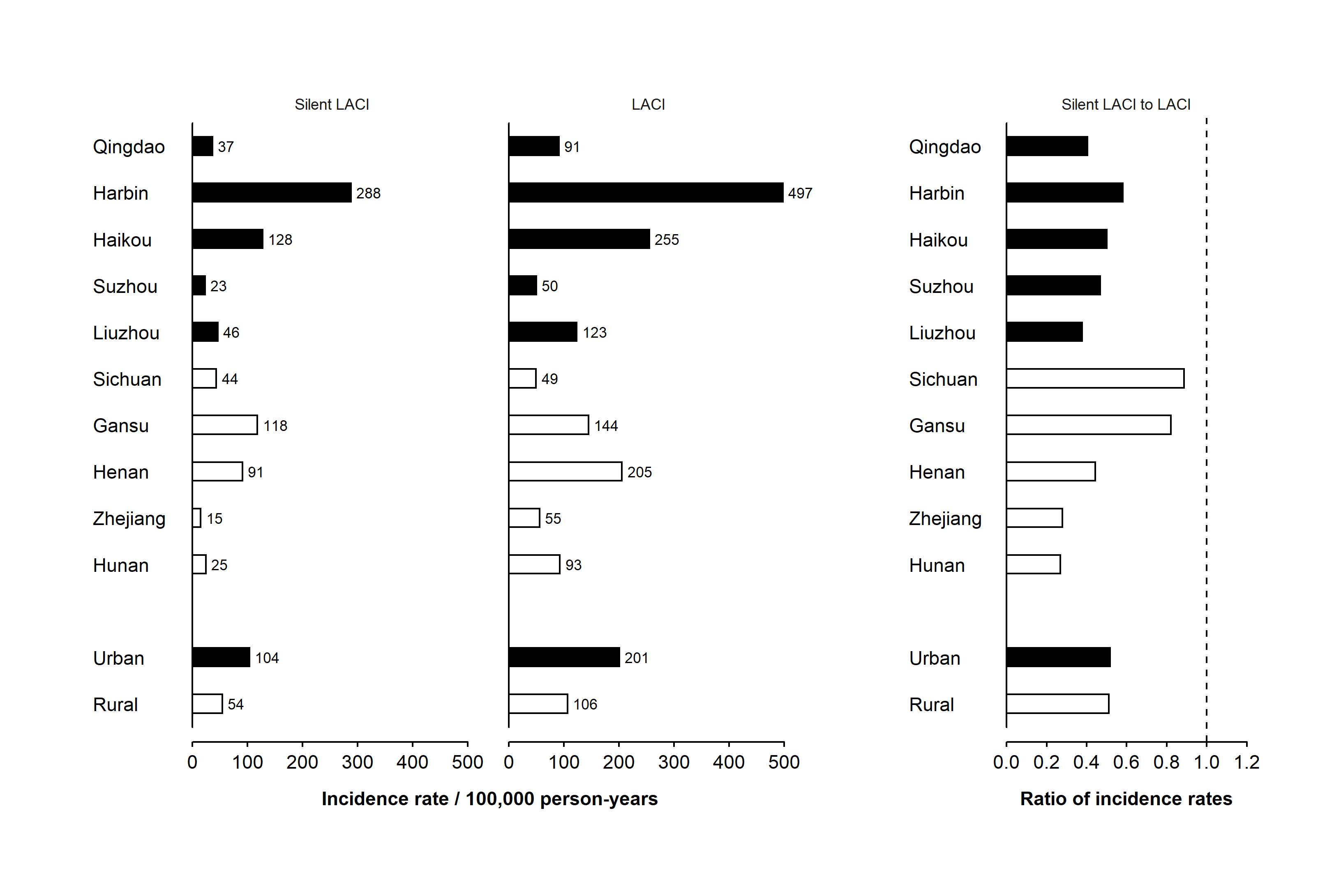
Supplementary Figure 2: Age−standardised event rate of silent LACI and LACI across 10 regions

# Supplementary Figure 3: Cumulative all−cause mortality rate from 28 days after first ischemic stroke of different subtypes by (a) sex, (b) area and (c) age


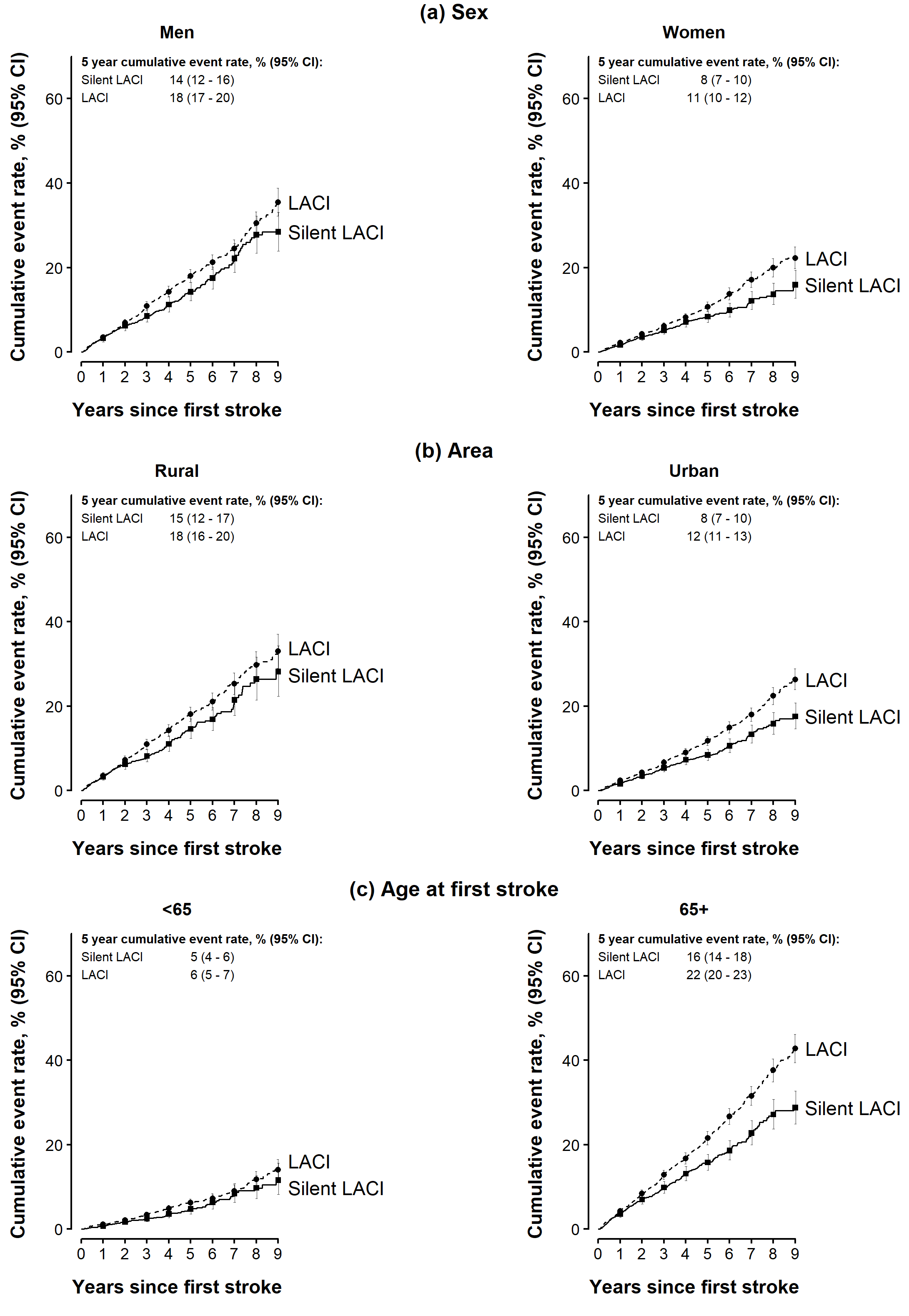


# Supplementary Figure 4: Cumulative event rate of recurrent stroke from 28 days after first ischemic stroke of different subtypes by (a) sex, (b) area and (c) age


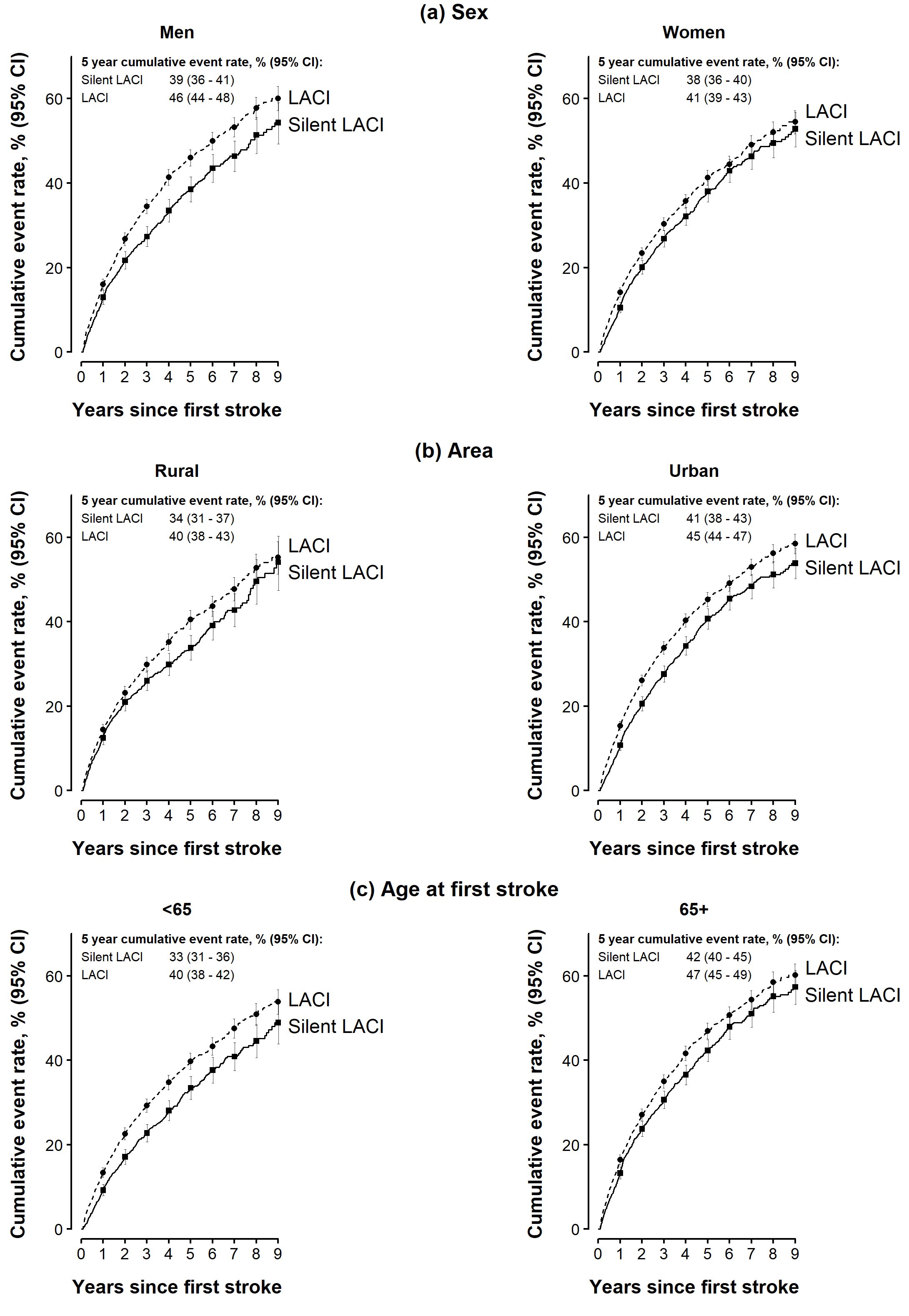


**Supplementary Figure 5: Cumulative event rate of (a) recurrent stroke and (b) all−cause mortality from 28 days after onset of first ischemic stroke event by imaging type**


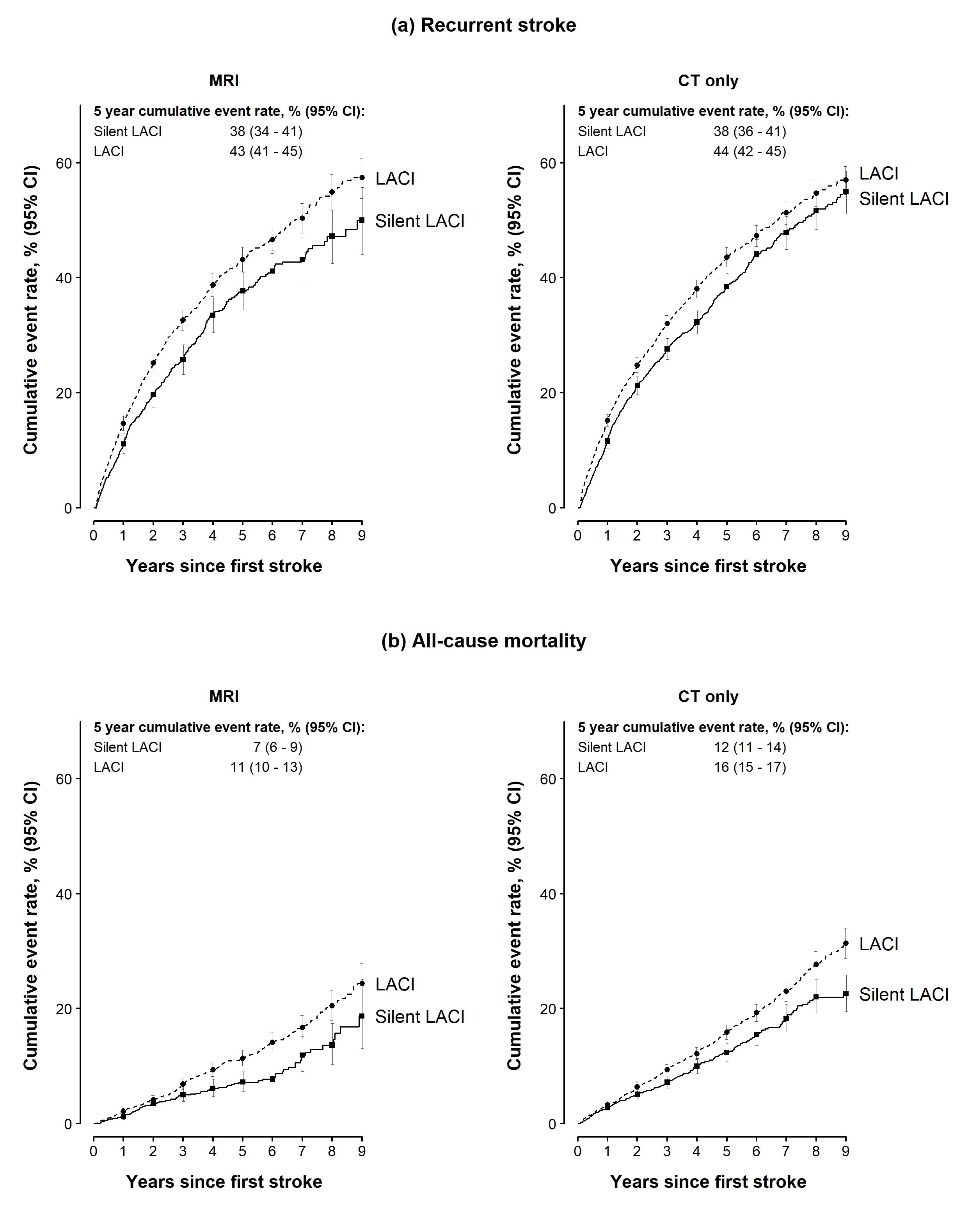

Supplement: Supplementary file 1 [file mmc1.docx]
